# A model for gene deregulation detection using expression data

**DOI:** 10.1186/1752-0509-9-S6-S6

**Published:** 2015-12-09

**Authors:** Thomas Picchetti, Julien Chiquet, Mohamed Elati, Pierre Neuvial, Rémy Nicolle, Etienne Birmelé

**Affiliations:** 1 Laboratoire MAP5, Université Paris Descartes and CNRS, Sorbonne Paris Cité, 45 rue des Saints-Pères, 75270 Paris Cedex 06, France; 2 Laboratoire de Mathématiques et Modélisation d'Evry (LaMME), Université d'Evry-Val-d'Essonne/UMR CNRS 8071/ENSIIE/USC INRA, Evry, France; 3 institute of Systems and Synthetic Biology (iSSB), CNRS, University of Evry, France; 4 Institut Curie, PSL Research University, UMR 144 75248 Cedex 05, France, CNRS 75248 Paris Cedex 05, France

**Keywords:** regulatory network, belief propagation, EM algorithm, deregulation, inference

## Abstract

In tumoral cells, gene regulation mechanisms are severely altered. Genes that do not react normally to their regulators' activity can provide explanations for the tumoral behavior, and be characteristic of cancer subtypes. We thus propose a statistical methodology to identify the misregulated genes given a reference network and gene expression data.

Our model is based on a regulatory process in which all genes are allowed to be deregulated. We derive an EM algorithm where the hidden variables correspond to the status (under/over/normally expressed) of the genes and where the E-step is solved thanks to a message passing algorithm. Our procedure provides posterior probabilities of deregulation in a given sample for each gene. We assess the performance of our method by numerical experiments on simulations and on a bladder cancer data set.

## Background

Various mechanisms affect gene expression in tumoral cells, including copy number alterations, mutations, modifications in the regulation network between the genes. A simple strategy to identify genes affected by these phenomena is to perform differential expression analysis. Results can then be extended to the scale of pathways using enrichment analysis [1] or functional class scoring [2]. However, such a strategy is blind to small variations in gene expression, especially as multiple testing correction applies. Moreover, it does not take interdependence between genes into account and can mark an expression change as abnormal when actually it is induced by a change in the regulators' activity. To overcome these drawbacks, an alternative strategy is to identify the affected genes by pointing important changes in the gene regulatory network (GRN) of the tumoral cell. Such an approach furthermore corresponds to the modelisation of phenomena altering regulation, as for instance mutations in regulatory regions [3].

The first step towards this is to procure a GRN. It can be obtained from curated databases or, in order to obtain tissue or condition-specific networks, reconstructed from expression data. In the latter case, the inference can be done by relying either on discrete or continuous models. In the discrete framework, gene expression profiles are discretized into binary or ternary valued variables (under-expressed/normal/over-expressed). The regulation structure is then given by a list of truth tables [4]. This approach allows in particular to take coregulation into account, that is to require the activity of a whole set of co-activators or co-inhibitors to activate or inhibit the target [5,6]. In the continuous case, inference can be done in a regression framework, where the expression of each target gene is explained by all its potential regulator genes. An edge is drawn between two genes if the corresponding regression coefficient is significantly different from zero, which can be deciphered by performing variable selection in the regression model. A popular choice for this task is to rely on sparsity-inducing penalties like the Lasso and its by-products [7,8]. In particular, some variants allow to account for co-regulation by favoring predefined groups of regulators acting together in a sign-coherent way [9]. Other forms of penalties encourage a predefined hierarchy between the predictors [10], *i.e*. the regulator genes in the case at hand.

To unravel deregulated genes by means of GRN, a first possibility is to infer several networks independently (one for each tissue) and to compare them. However, due to the noisy nature of transcriptomic data and the large number of features compared to the sample size, most of the differences found in the networks inferred independently may not be linked with underlying biological processes. Methods have therefore been developed to infer several networks jointly to share similarities between the different tissues and penalize the presence of an edge in only one of them. Such methods exist for both time series [11] or steady-state [12] data.

A second possibility is to assess the adequacy of gene expression in tumoral samples to a reference GRN, in order to exhibit the more striking discrepancies - *i.e*. the regulations which are not fulfilled by the data. In this perspective, [13] use an heuristic in a Boolean framework to update the regulatory structure by minimizing the discrepancies between the reference GRN and a new data set. A similar approach is depicted in [14] to predict the discrepancies and the unobserved genes of the network. More methods analyzing the coherence between known signaling pathways and gene data sets can be found in the review [15]. Still, they focus on checking the validity of the network rather than highlighting genes with an abnormal behavior.

At the pathway level rather than the gene level, it is possible to look for sample- specific regulation abnormalities by using SPIA [16]. PARADIGM [17] generalizes SPIA on heterogeneous data (DNA copies, mRNA and protein data). Moreover, it determines a score of activity for each gene of a pathway for each sample of the data set, and the use of hidden variables allows to compute this score even if some of the genes of the pathway are not measured. The method is however not network-wide in the sense that each gene has a deregulation score by pathway it belongs to, and pathways are treated independently. Moreover, as the pathways are extracted from curated databases, the regulations taken into account are not tissue-specific.

The aim of this paper is to develop a methodology to provide a network-wide deregulation score for each gene and each sample by taking the whole regulation network into account. For this purpose, we introduce a model based on a regulatory process in which genes are allowed to be deregulated, *i.e*. not respond to their regulators as expected. An EM strategy is proposed for parameter inference, where the hidden variables correspond to the status (under/over/normally expressed) of the genes. The E-step is solved thanks to a message passing algorithm. At the end of the day, the procedure provides *posterior *probabilities of deregulation in a given sample for each target gene. We assess the performance of our method for detecting deregulations on simulated data. We also illustrate its interest on a bladder cancer data set, where we study the deregulations according to two reference GRN obtained by two state-of-the-art network inference procedures on a consensus expression data set.

## Methods

### The model

Our model draws inspiration from LICORN [5], a model originally developed for network inference purposes. LICORN considers a regulation structure in which genes are either regulators (transcription factors - TFs) or target genes. The expressions are discretized and each gene *g *is characterized by a ternary value *S_g _∈ {−*1, 0, +1*} *encoding its expression status - under-, normally, or over-expressed. The regulation of each target gene *g *is governed by a set of co-activators *A*(*g*) and co-inhibitors *I*(*g*) among the TFs. Those sets are endowed with some "collective status" described by variables $S_{g}^{{}^{A}}$ and $S_{g}^{I}$, assuming that regulation works in a cooperative way: hence, the collective state of a set of regulators is over- (resp. under-) represented if and only if all elements in the set share the same status. Finally, the status *S_g _*of the target gene *g *is deduced from $S_{g}^{{}^{A}}$ and $S_{g}^{I}$ by following Truth Table 1.

**Table 1:** LICORN truth table.

	Activator state		
	
Inhibitor state	-	0	+
-	0	+	+

0	-	0	+

+	-	-	-

How the target gene behaves (unless it is deregulated) according to its co-activators' state *A *and co-inhibitors' state *I*.

In order to detect deregulated target genes given a regulatory network and gene expression profiles, we apply two major modifications to the LICORN model: first, we avoid discretization of the data by considering all the ternary variables introduced so far as hidden random variables. The expression *X_g _*of a gene *g *is assumed to follow a normal distribution with parameters that depend on the hidden status, *i.e*., *X_g _|S_g _*= *s ~ N *(*µ_s_, σ_s_*). Second, we introduce for each gene an indicator variable *D_g _*for deregulation, such that *D_g _*= 1 with probability Ε. Renaming the result of the truth table by $S_{g}^{R}$, the final status of the target is then deduced from the values of *D_g _*and $S_{g}^{R}$:

$$\left\{\begin{array}{cc} S_{g}=S_{g}^{R} & \text{if}\;D_{g}=\;0,\\ \forall s\neq S_{g}^{R},\mathbb{P}\left(S_{g}=s\right)=\frac{1}{2} & \text{if}\;D_{g}=\;1. \end{array}\right)$$

For completeness, we must specify the distribution of the hidden states *S_g _*for each TF: we assume independent multinomial distributions with parameters ***α ***= (*α_−_, α*_0_*, α*_+_).

**Figure 1:**
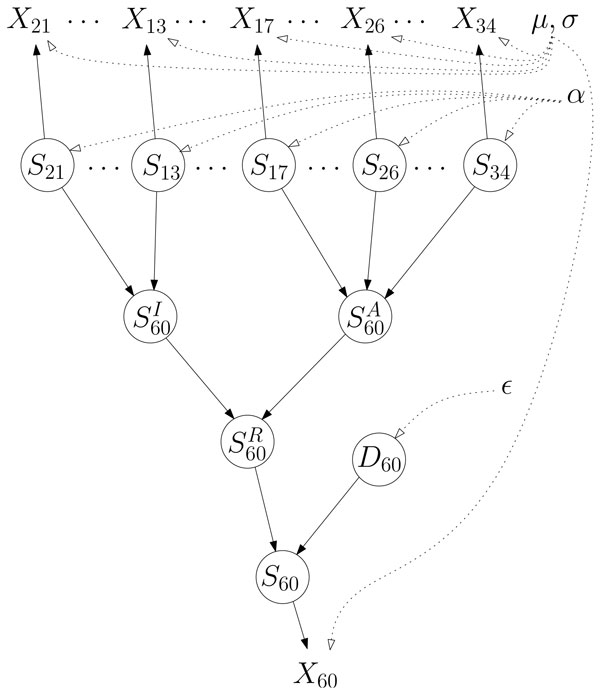
**The model for one target gene regulated by two co-inhibitors and three co-activators**. The circled variables are hidden. A dashed edge indicates that the distribution of the variable depends on the corresponding parameter.

The model is summarized for one target gene in Figure 1. For the sake of conciseness, the vector ***θ ***entails all parameters of the models, that is, the means and standard deviations of the Gaussians, the vector ***α ***of proportions and the deregulation rate Ε. The data set contains *n *samples, *r *TFs and *t *target genes. We denote by **Z **the *n × *(*r *+ 5*t*) matrix of all hidden states and by **X **the *n × *(*r *+ *t*) matrix of all expression variables.

Note that the dependencies among variables are acyclic, implying that the likelihood can be decomposed in a product.

$$p\left(X,Z|\theta \right)=\prod p\left(S_{j}|\alpha \right)\times \prod p\left(S_{i}^{A}|S_{j}\ldots \right)\times \prod p\left(S_{i}^{I}|S_{j}\ldots \right)\times \prod p\left(S_{i}^{R}|S_{i}^{I},S_{i}^{A}\right)\times \prod p\left(D_{i}|E\right)\times \prod p\left(S_{i}|S_{i}^{R},D_{i}\right)\times \prod p\left(X_{k}|S_{k},\mu ,\sigma \right)$$

For sake of readability, the indices of the products are omitted in the above formula. However, it should be clear when the product runs over target genes, regulator genes or all of them.

### Estimation algorithm

As usual with latent variable models, the likelihood is intractable as the number of potential states of the hidden variables grows exponentially with the number of variables. Therefore, we adopt an EM-like strategy [18] by iterating the following steps, starting from an initial guess ***θ***^0 ^of the model parameters:

**E-step: **Fix ***θ ***and compute the conditional probability distribution of the hidden variables, given the observed expression values: q(Z)=ℙ(Z|X,θ)

**M-step: **Fix *q *and find ***θ ***that maximizes $\sum q\left(Z\right)\text{log}\mathbb{P}\left(X,Z|\theta \right)$

*Step E*. The first issue at stake in the E-step is to deal with the number of potential states for the hidden variables of all the genes. Fortunately, we only need their marginal distributions in the M step, as will be shown in the corresponding section. Still, we need a way to compute these marginals without having to compute the joint distribution first.

To handle this issue, we rely on Belief Propagation [19] - a.k.a *message-passing algorithm *- to perform the E step, since the probability distribution arising from our model is easily represented as a factor graph. Indeed, consider a set of discrete values for all variables $S_{g}^{{}^{A}}$, $S_{g}^{{}^{I}}$, $S_{g}^{{}^{R}}$ and *D_g _*. Conditionally on **X**, the probability for the discrete variables to match the given value is proportional to the product of the following factors:

1. *α_Sg _*for each regulator gene *g ∈ R*;

2. *E *if *D_g _*= 1, and $\frac{1-E}{2}$ if *D_g _*= 0, for each target gene *g ∈ T*;

$\frac{1}{\sigma }\text{exp}\frac{-{\left(X_{g}-\mu \right)}^{2}}{2\sigma^{2}}$ for each gene *g ∈ G *(regulator or target), where *µ *and *σ *are the mean expression and standard deviation associated to state *S_g _*;

4. a factor equal to one if $S_{g}^{{}^{A}}$ correctly represents the collective state of *g*'s activators, and zero otherwise;

5. a factor equal to one if $S_{g}^{{}^{I}}$ correctly represents the collective state of *g*'s inhibitors, and zero otherwise;

6. a factor equal to one if $S_{g}^{{}^{R}}$ is the entry in Table 1 corresponding to $S_{g}^{{}^{A}}$ and $S_{g}^{{}^{I}}$, and zero otherwise;

7. a factor equal to one if either *D_g _*= 0 and *S_g _*= $S_{g}^{{}^{R}}$ or *D_g _*= 1 and $S_{g}\neq S_{g}^{{}^{R}}$, and zero otherwise.

**Figure 2:**
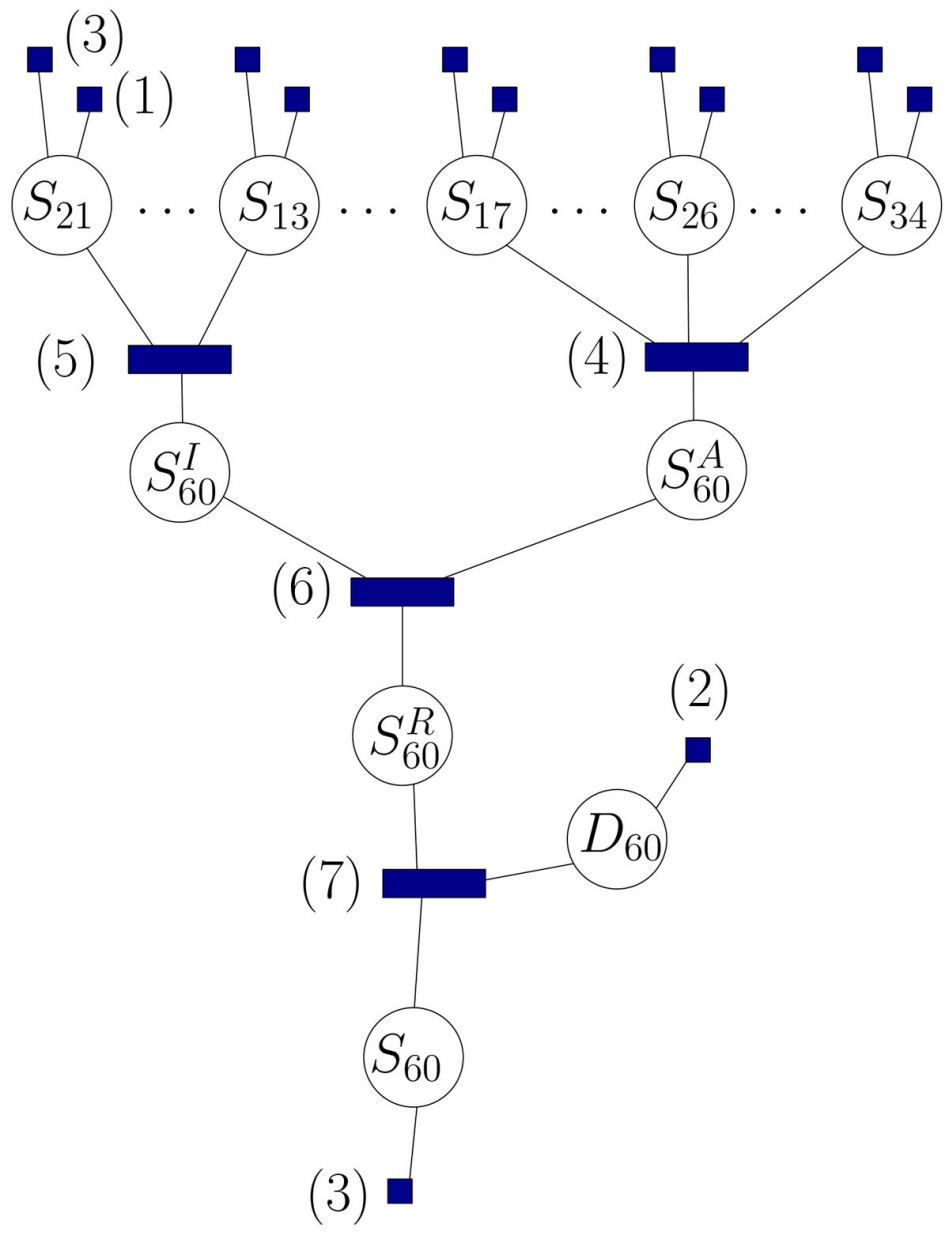
**A partial view of the factor graph**. The factor graph corresponding to Figure 1. The rectangles correspond to the factors, and are numbered according to the text. The algorithm iteratively updates the distribution of the circled variables.

This factorization translates into the factor graph depicted in Figure 2 (a graph whose nodes are the variables and the above factors, each factor being connected to the variables it depends on). We use the *SumProduct *Belief Propagation algorithm, implemented in the Dimple library [20] to compute approximated marginals of every hidden variable, given the regulation network, the parameter set, and the expression values. In the case where multiple samples are given, this can be done separately for each one since the samples are considered as independent.

*Step M*. In this step we keep the probability distribution *q *fixed and look for the parameters ***θ ***that maximize

$$\underset{Z}{\sum }q\left(Z\right)\;\text{log}\;\mathbb{P}\left(X,Z|\theta \right)$$

Since ℙ(X,Z|θ) is a product of simple factors, its logarithm is the sum of these factors. Also, note that boolean factors (4-7) can be omitted since they have no effect on the sum: whenever $q\left(Z\right)\neq 0$, these factors must be equal to 1 hence the logarithm is 0.

Calling *G *the set of genes, *R ⊂ G *the set of regulators and *T ⊂ G *the set of target genes, we are left to maximize the sum over all samples of

$$\begin{array}{c} \underset{g\in R}{\sum }\underset{Z}{\sum }q\left(Z\right)\;\text{log}\alpha_{S_{g}}\\ +\underset{g\in T}{\sum }\underset{Z}{\sum }q\left(Z\right)\left(D_{g}\text{log}E+\left(1-D_{g}\right)\text{log}\frac{1-\varepsilon }{2}\right)\\ \;+\underset{g\in G}{\sum }\underset{Z}{\sum }q\left(Z\right)\left(\frac{-{\left(x_{G}-\mu_{S_{g}}\right)}^{2}}{2\sigma_{S_{g}}^{2}}-\text{log}\sigma_{S_{g}}\right) \end{array}$$

These three terms depend on separate parameters and can be maximized separately. Moreover, we only require the marginals of variables *S_g _*and *D_g _*for this task, and not the full distribution *q*. Denoting by *I *the set of samples, it is straightforward to show that the former sum is maximized for the following parameters:

$$\begin{array}{c} \alpha_{-}\propto \underset{i\in I}{\sum }\underset{g\in R}{\sum }q\left(S_{i,g}=-1\right),\alpha_{0}-\propto \underset{i\in I}{\sum }\underset{g\in R}{\sum }q\left(S_{i,g}=0\right),\alpha_{+}\propto \underset{i\in I}{\sum }\underset{g\in R}{\sum }q\left(S_{i,g}=+1\right),\\ \;\varepsilon \propto \underset{i\in I}{\sum }\underset{g\in T}{\sum }q\left(D_{i,g}=1\right),\;(1-\varepsilon )\propto \underset{i\in I}{\sum }\underset{g\in T}{\sum }q\left(D_{i,g}=0\right),\\ \;\mu_{s}=\frac{\sum_{i}\sum_{g}q\left(S_{i,g}=S\right)X_{i,g}}{\sum_{i}\sum_{g}q\left(S_{i,g}=S\right)},{\;\sigma }_{s}^{2}=\frac{\sum_{i}\sum_{g}q\left(S_{i,g}=S\right){\left(\mu_{s}-X_{i}\right)}^{2}}{\sum_{i}\sum_{g}q\left(S_{i,g}=S\right)} \end{array}$$

### Complexity analysis

Step M only involves computing a few sums of size [number of genes]*×*[number of samples] and is not time-consuming. Step E performs for each sample a fixed number of passes of Belief Propagation in the factor graph. Each pass consists in updating every node with information from its neighbors. The complexity of updating a factor grows exponentially with its degree, therefore it is important to limit the number of variables of each factor. It is done by replacing the factors corresponding to the types (4) and (5) in Figure 2 by tree-like structures with many factors having 3 variables each.

With this approach the graph has approximately *N *= 2*E *+ *G *nodes, where *E *is the number of regulator-target edges in the regulation network, and *G *the number of genes. A personal computer performs a few million node updates per second, thus step E will run in *t *seconds if *N ×*[number of passes]*×*[number of samples] is not much greater than *t *millions.

### Regulatory network inference from expression data

To apply our methodology to real data, we use two different inference methods.

*LICORN*. The first one, named hLICORN, corresponds to the LICORN model and is available in the CoRegNet Bioconductor package [6]. In a first step, it efficiently searches the discretized gene expression matrix for sets of co-activators and co-repressors by frequent items search techniques and locally selects combinations of co-repressors and co-activators as candidate subnetworks. In a second step, it determines for each gene the best sets among those candidates by running a regression. hLICORN was shown to be suitable for cooperative regulation detection [5,6].

*Cooperative-Lasso + Stability Selection*. The second inference procedure applies in a continuous setup. It consists in two steps: first, a selection step performed with a sparse procedure; and second, a resampling step whose purpose is to stabilize the selection for more robustness in the reconstructed network. Here are some details.

*Step 1: selection*. For each target gene, a sparse penalized regression method is used to select the set of relevant co-activators and co-inhibitors among all possible transcription factors. When no special structure is assumed in the network, this task can be performed with the Lasso penalty, as it was successfully applied for network inference in [8]. Here, however, we are looking for sets of regulators that work group-wise, either as co-activators or co-inhibitors. To favor such a structure, we build on the penalty proposed in [12,9] that encourages selection of predefined groups of variables sharing the same sign (thus being either co-activators or co-inhibitors). This regularization scheme is known as the "cooperative-Lasso". It was originally designed to work with a set of groups that form a partition over the set of regulators. Here, we extend this method to a structure that defines a hierarchy (or tree) on the set of regulators *R *. We denote by $H=\left\{H_{\text{1}},\;.\;.\;.\;,\;H_{K}\right\}$this structure, with $H_{k}$ the *k*th (non-empty) node of the hierarchy.

Technically, the optimization problem solved for selecting regulators of gene *g *is the following penalized regression problem

$${\hat{\beta }}^{\left(g\right)}=\underset{\beta^{\left(g\right)}\in {\mathbb{R}}^{\left|R\right|}}{\text{arg min}}\frac{1}{2}{\left\|X_{g}-X_{R}\beta^{\left(g\right)}\right\|}^{2}+\lambda \sum_{k=1}^{K}{\left\|{\left(\beta_{{ℋ}_{k}}^{\left(g\right)}\right)}^{+}\right\|}_{2}+{\left\|{\left(\beta_{{ℋ}_{k}}^{\left(g\right)}\right)}^{-}\right\|}_{2},$$

with **X***_g _*the expression profile of gene *g *and **X***_R _*the expression profiles of the regulators. The parameter *λ >*0 tunes the amount of regularization, and thus the number of regulators associated with gene *g*; **v**^+ ^and **v***^− ^*are the positive, respectively the negative elements of a vector **v**, and $v_{{ℋ}_{k}}$ the restriction of **v **to the elements in node $H_{k}$ of the hierarchy. Hence, this penalty favors selection of sign-coherent groups of variables, like ${\left(\beta_{H_{k}}^{{}^{\left(g\right)}}\right)}^{+}$, standing for the estimated co-activators of gene *g *in node $H_{k}$ of the hierarchy, or ${\left(\beta_{H_{k}}^{{}^{\left(g\right)}}\right)}^{-}$, the corresponding co-inhibitors.

*Step2: Stabilization*. We fit a sparse model as described above for each target gene, regressing on the same set of regulators *R*. The hierarchy ℋ that we used is obtained by performing hierarchical clustering with average linkage on a distance based upon the correlation between expression profiles. We use the same *λ *for each gene, which is chosen large enough in order to select at least one set of regulators for all target genes. To select the final edges in the network, we rely on the stability selection procedure of [21], which was successfully applied to the reconstruction of robust regulatory networks in the case of a simple Lasso penalty [7], and is known to be less sensitive than selecting one *λ *per gene (*e.g*. by cross-validation). This technique consists in refitting the regression model on many subsamples obtained by drawing randomly *n/*2 observations from the original data set. We replicate 10,000 times this operation and obtain an estimated probability of selection for each edge. We fix the threshold in order to select a number of edges similar to LICORN, which corresponds to edges with a probability of selection greater than 0.65.

## Results and discussion

### Classification performances on simulated data sets

In our experiments, the score *q*(*D_i,g _*= 1) is used to determine if gene *g *is deregulated or not in sample *i*. Performances are evaluated with Precision-Recall (PR) curves, which are known to be more informative than ROC curves or accuracy [22] when considering classification problem with very imbalanced data sets.

We generate expression data sets according to the model described earlier and feed them to the EM algorithm to evaluate its performance. To study the impact of each parameter, we try several values of this parameter while all others remain fixed to their default value. Ten data sets are generated and processed in each setting, resulting in 10 PR curves. We thus obtain clouds of curves, measuring both the variability for a given parameter set and the influence of the varying parameter.

**Figure 3:**
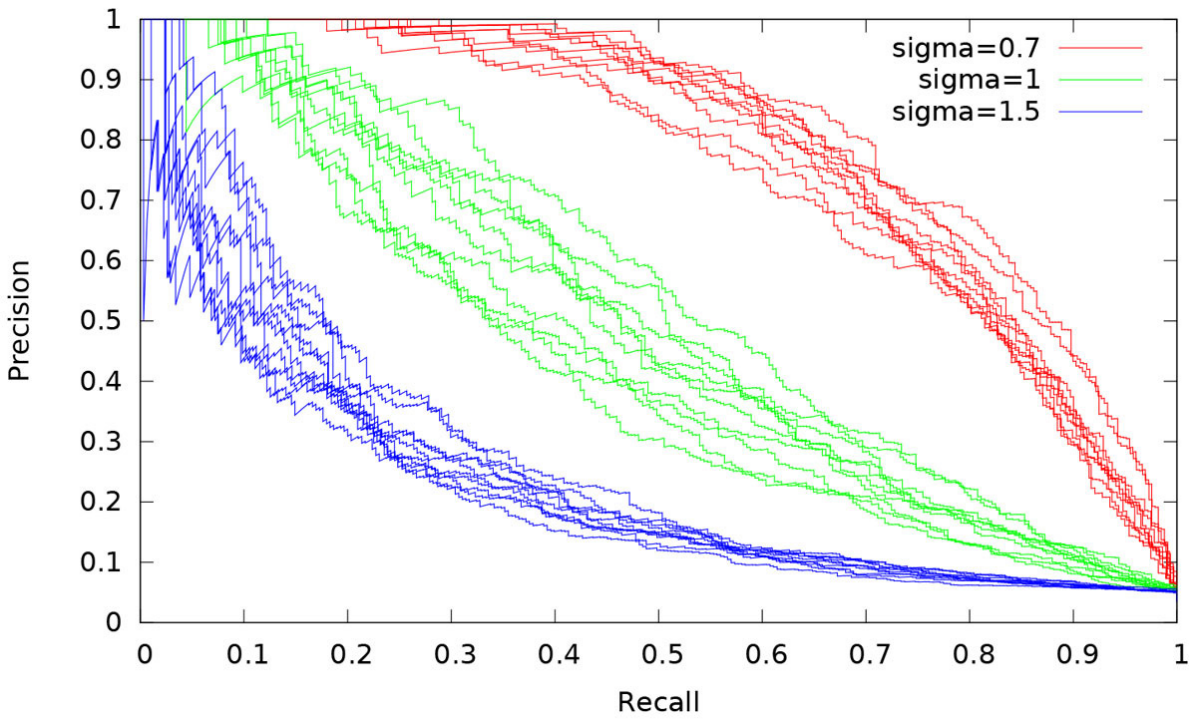
**Influence of *σ***. PR curves for simulations with varying *σ*, with means (*µ_−_, µ*_0_*, µ*_+_) = (*−*1, 0, 1). Ten simulations are run for each value.

We unsurprisingly note that *σ *has dramatic effect (see Figure 3). As a rule of thumb to distinguish two states from one another, the associated standard deviations must be smaller than the difference between their mean expressions.

**Figure 4:**
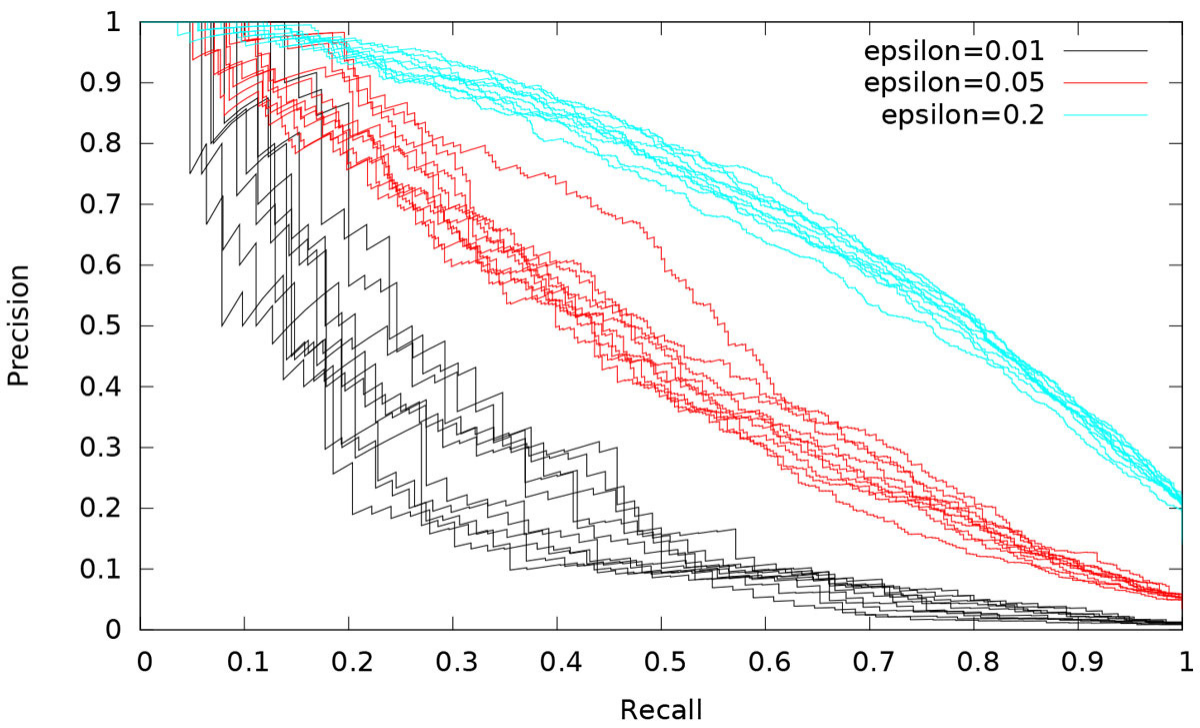
**Influence of Ε**. PR curves for simulations with varying Ε. Ten simulations are run for each value.

Meanwhile, large values of *E *mechanically result in better PR: the more the deregulated genes, the more the true positives among all positives (Figure 4).

On the contrary, all other parameter have little effect on the performance and we thus postpone the associated PR curves to the Additional File 1. Those parameters are *µ, α*, the number of passes in the Belief Propagation algorithm (as long as it is greater than five), the number of genes and the sample size (as long as their product is of several hundreds).

### Managing the False Discovery Rate

Consider couples (*i, g*) whose deregulation score *q*(*D_i,g _*= 1) = *s*: this score being a *posterior *probability, the expected proportion of true (respectively false) positives is *s *(respectively 1 *− s*). Similarly, if *K *pairs pass the threshold, the expected number of true positives among them is the sum of their scores, denoted by *S*. The false discovery rate (FDR) may be estimated by (*K − S*)*/K*. In practice, aiming for a particular FDR, one can start with a threshold of 1 and lower it gradually: as more pairs get selected, the ratio (*K − S*)*/K *gradually increases. All one has to do is stop when it reaches the intended FDR. The concordance between the intended FDR and the actual proportion of false positives is illustrated on simulated data sets in the Additional File 1.

### Tests on real data

We applied our method to the bladder cancer data set available in the R-package CoRegNet [6]. Expression data from patients with different status was pooled to infer gene co-regulatory networks with two independent procedures, namely *hLICORN *and the hierarchical *Cooperative-Lasso*. The inferred networks reflect the regulation trends over the whole set of 184 samples. Our EM algorithm is then run using the same expression data, but since samples are now treated individually, the results reflect how each sample violates the regulatory rules generally followed by the others.

On real data, the true deregulation status is unreachable. Hence, we match our result with Copy Number Alteration (CNA) data collected from the same samples, in order to support that our method correctly identifies deregulated gene-sample pairs. We do not expect CNAs to precisely coincide with failures of the regulation network, so we do not hope to detect exactly those pairs that present a CNA. However, the number of gene copies influences the expression independently from expression of the TFs [23]. We therefore expect to observe a link between CNA and gene deregulations.

**Figure 5:**
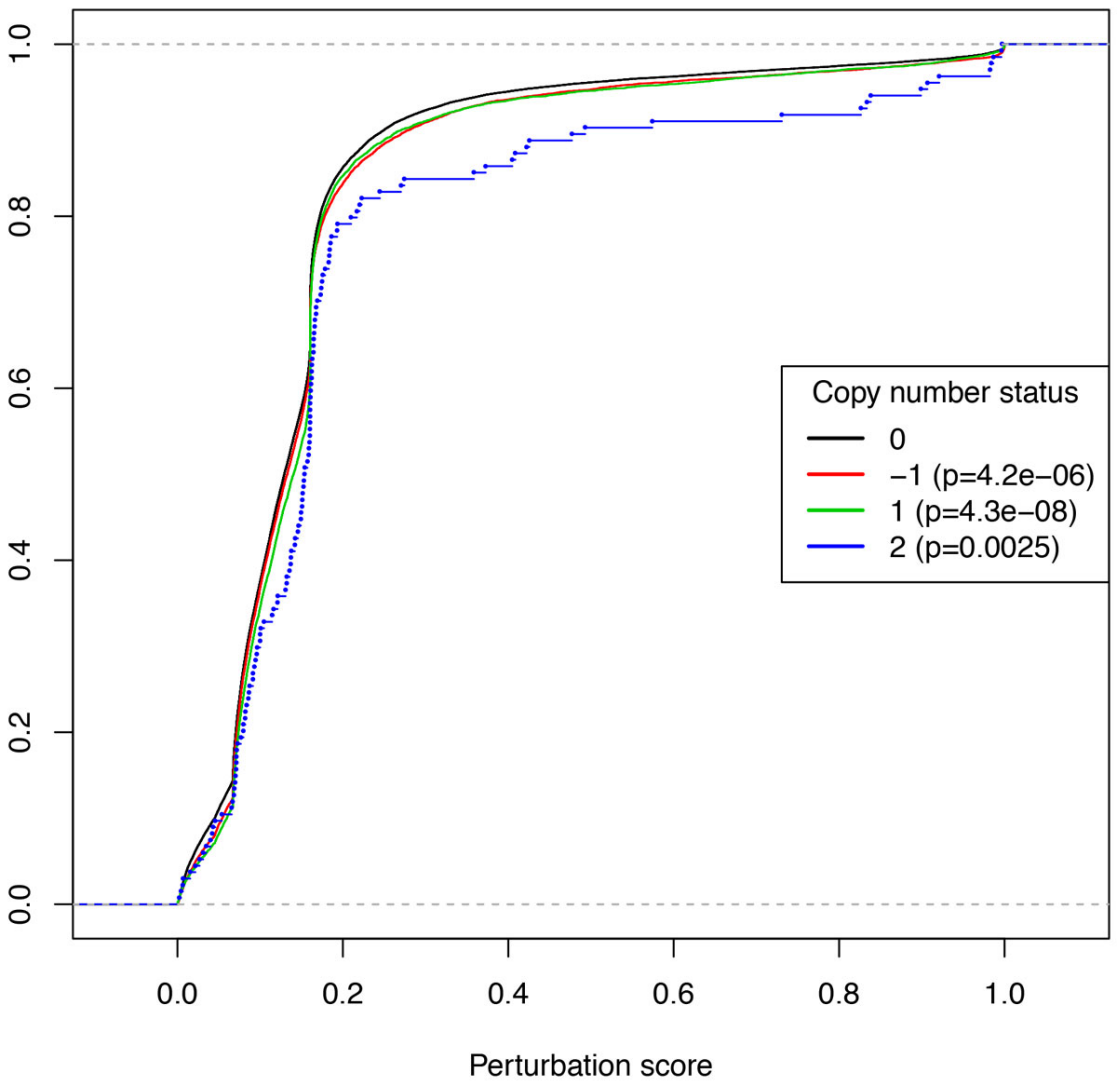
**Empirical cumulative distribution of scores, by Copy-Number status**. Student's test is used to compare every altered state with the normal.

To this end, we use CNA data provided by the CoRegNet package, associating to each gene-sample pair a copy number state: 0 for the diploid state (two copies), 1 for a copy number gain, *−*1 for a copy number loss, and 2 for a copy number amplification. Figure 5 compares the distribution of the perturbation scores across copy number states by representing, for each copy number class, the empirical cumulative distribution function of the perturbation scores. For each value *s *of the perturbation score in abscissa, the ordinate is the proportion of gene-sample pairs with a score greater than *s*. The fact that the curve corresponding to the diploid state is above all the other curves indicates that gene-sample pairs having a CNA are given a higher perturbation score than diploid gene-sample pairs by our deregulation model. Although the difference seems slight, it is highly significant given the large number of scores, as indicated by the *p*-value of the Student test for the pairwise differences between the diploid state and each of the other altered states. As expected, the scores of the "amplification" state 2 are also higher than the scores of "gain" state 1.

## Conclusion

In the present article, we develop a statistical model for gene expression based on a hidden regulatory structure. Given a reference GRN, it allows to determine which genes are misregulated in a sample, meaning an expression which does not match the network given the expression of its regulators. Numerical experiments validate the algorithmic procedure: when applied to bladder cancer data with known CNA, the deregulation score is higher in samples in which genes have an altered number of copies.

We believe that our methodology will be useful to understand which regulation mechanisms are altered in different cancer subtypes. Indeed, the results of our methodology are sample-specific. However, characterizing the deregulations which are common to most of the individuals suffering a given cancer subtype is a promising perspective.

The integration of CNA to the methodology, as already done in the context of differential expression [24], will also be considered in future work, as it would allow a better power for detecting genes suffering misregulation due to a copy alteration.

## Availability of supporting data

The EM algorithm described in this article is available as a Java archive at http://www.math-info.univ-paris5.fr/~ebirmele/index.php?choix=6/

Bladder cancer data and hLicorn are available through the CoRegNet Bioconductor package.

## Abbreviations

CNA: Copy Number Alteration GRN: Gene Regulatory Network PR curve: Precision-Recall ROC curve: Receiver Operating Characteristic curve TF: Transcription factor

## Competing interests

The authors declare that they have no competing interests.

## Authors' contributions

The work presented here was carried out in collaboration between all authors. ME and EB conceived the study. TP and EB designed it and wrote the manuscript. JC, PN and RN brought their expertise on inference and statistical interpretation on the real data. All authors provided valuable advises in developing the proposed method and modifying the manuscript. All authors read and approved the final manuscript.

## Supplementary Material

Additional File 1 File containing PR curves for varying *α, µ*, the number of genes/samples and the number of belief propagation iterations. It also contains figures illustrating the FDR estimation on simulated data. Click here for file

## Declarations

This work was partially supported the CNRS (CREPE, PEPS BMI). Publication charges were funded by CHIST-ERA grant (AdaLab, ANR 14-CHR2-0001-01).

This article has been published as part of *BMC Systems Biology *Volume 9 Supplement 6, 2015: Joint 26th Genome Informatics Workshop and 14th International Conference on Bioinformatics: Systems biology. The full contents of the supplement are available online at http://www.biomedcentral.com/bmcsystbiol/supplements/9/S6.
