# Supplementary material for: A model for gene deregulation detection using expression data
Source: BMC Syst Biol. 2015 Dec 9;9(Suppl 6):S6. doi: 10.1186/1752-0509-9-S6-S6 (PMC4674863; doi:10.1186/1752-0509-9-S6-S6)
Supplement: Additional File 1 — File containing PR curves for varying α, µ, the number of genes/samples and the number of belief propagation iterations. It also contains figures illustrating the FDR estimation on simulated data. [file 1752-0509-9-S6-S6-S1.pdf]

A model for gene deregulation detection using expression data

Supplementary Material

Influence of parameters on simulations

The method was tested on simulated datasets generated according to the described model. Default parameter values were :

- $\mu = (-1, 0, 1)$
- $\sigma = (1, 1, 1)$
- $\alpha = (0.2, 0.6, 0.2)$
- $\epsilon = 0.05$
- networks having 200 genes
- target genes have on average 3 co-activators and 3 co-inhibitors
- 40 expression samples
- 10 Belief Propagation passes in each step E

Each parameter was studied individually, making 10 simulations for each considered value of the parameter under study, while all the other parameters kept their default values. The starting point of the EM algorithm was always the following parameter set :

- $\mu = (-1, 0, 1)$
- $\sigma = (1, 1, 1)$
- $\alpha = (0.1, 0.8, 0.1)$
- $\epsilon = 0.1$

The value of μ was changed in two ways, scaling (fig 2) and translation (fig 3). For scaling, the values of σ were scaled by the same factor as μ. The starting point of the EM algorithm was kept the same.

Other quantities that have been studied in this way are the number of genes (fig 4), of samples (fig 5), and of belief propagation passes in each EM iteration (fig 6).

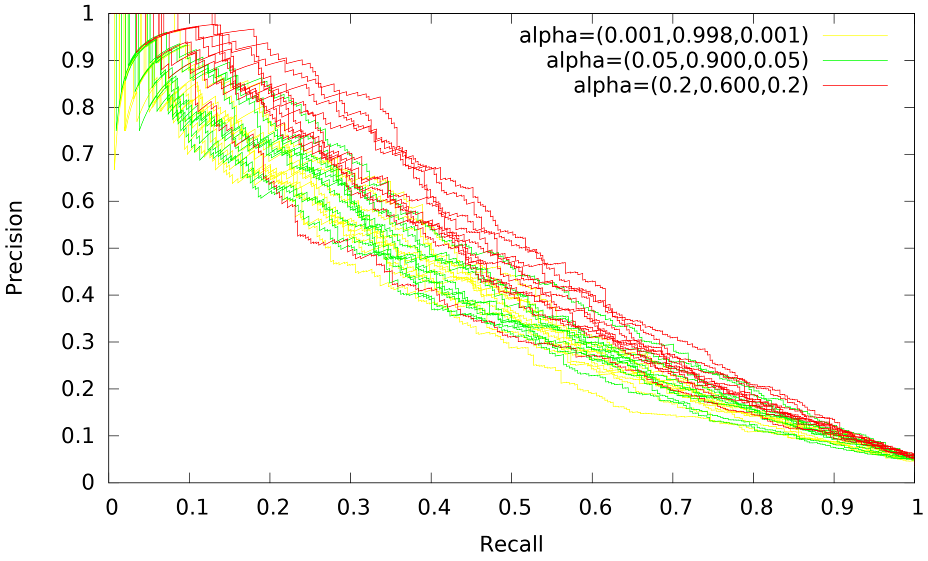

Figure 1: Varying α

Managing the FDR

When K scores are above a score threshold, we compute their sum S and use $(K-S)/K$ as an estimate of FP/K, the proportion of false positives among them. An approach to achieve FDR=x for a given x would be to set the threshold so that $(K-S)/K = x$. Fig 7 shows for ten simulations (made with the default parameter set) how the actual proportion of false positives relates to the FDR we aim for. There is high variablity near zero, because when we expect very few false positives, i.e. $K-S$ is small, one more or one less makes a big difference. Thus we study is how this variability evolves with the value of $K-S$: this is shown for the same ten simulations in fig 8, and helps to estimate, based on $K-S$, how precise this approach is in managing the FDR.

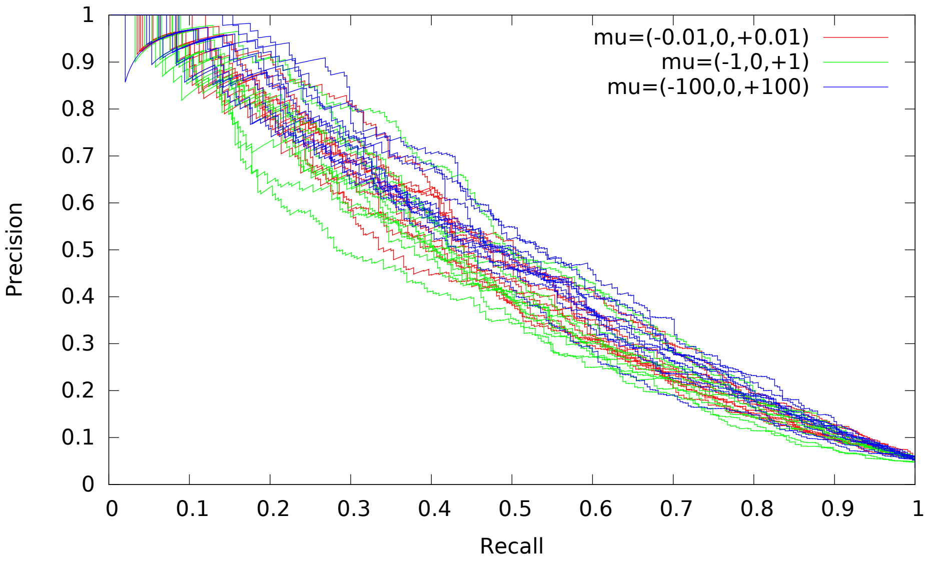

Figure 2: Scaling μ

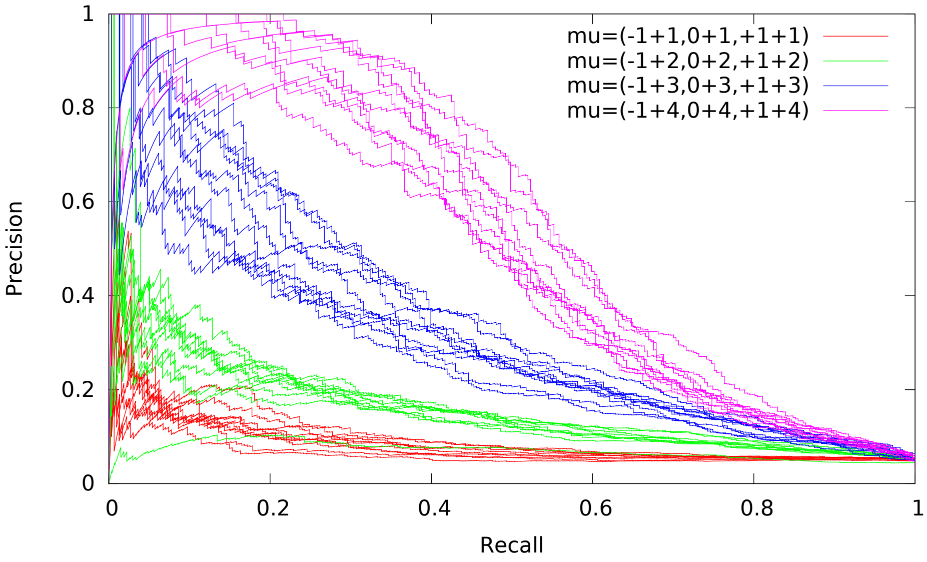

Figure 3: Translating μ

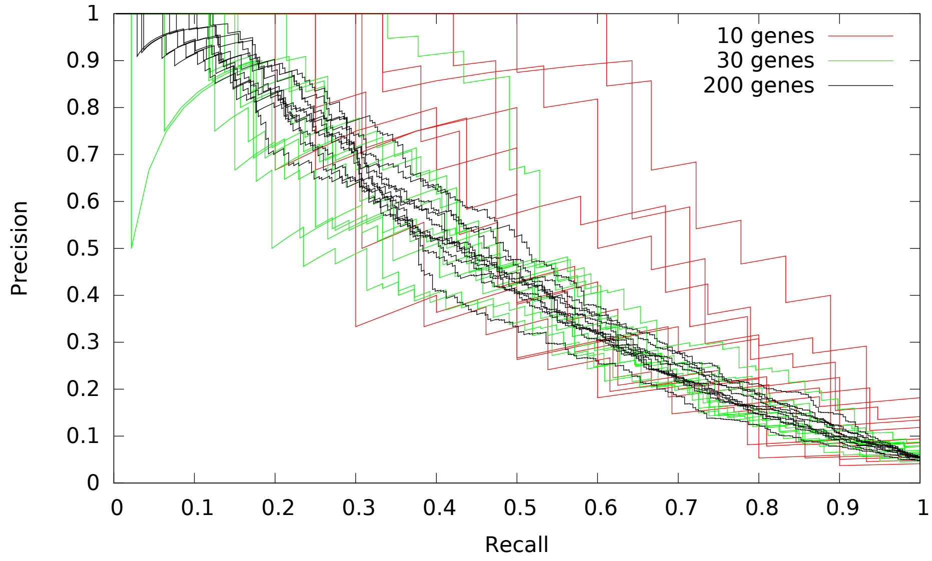

Figure 4: Changing the number of genes : fewer genes bring more variability but no loss in average performance

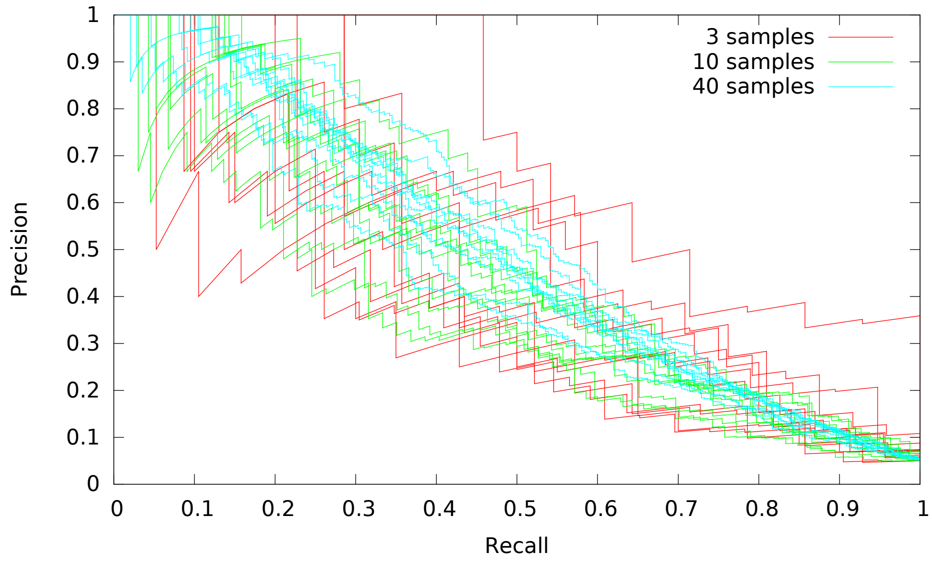

Figure 5: Changing the number of samples : fewer samples bring more variability but no loss in average performance

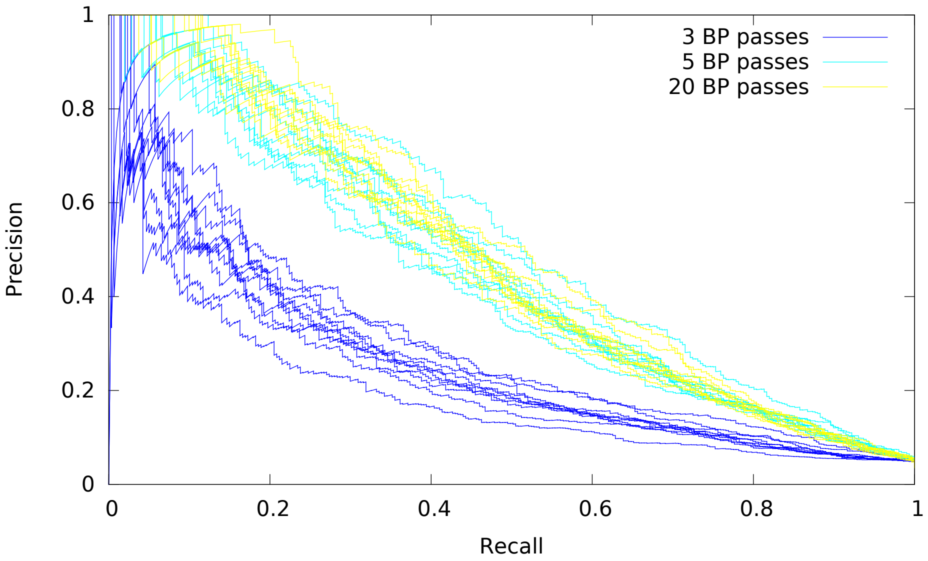

Figure 6: Changing the number of belief propagation passes : 5 passes are necessary to compute good results, but more passes does not result in better results.

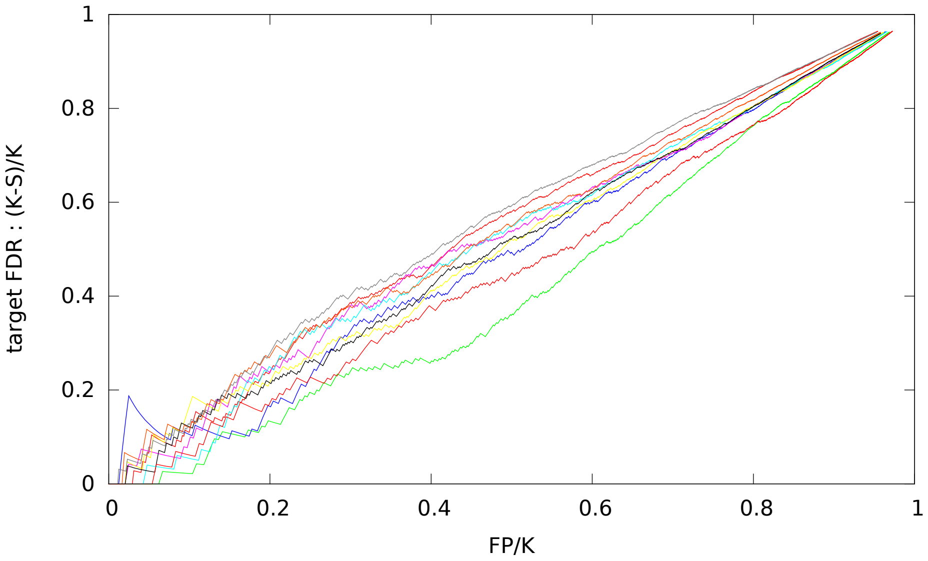

Figure 7: The actual proportion of false positives follows the intended FDR

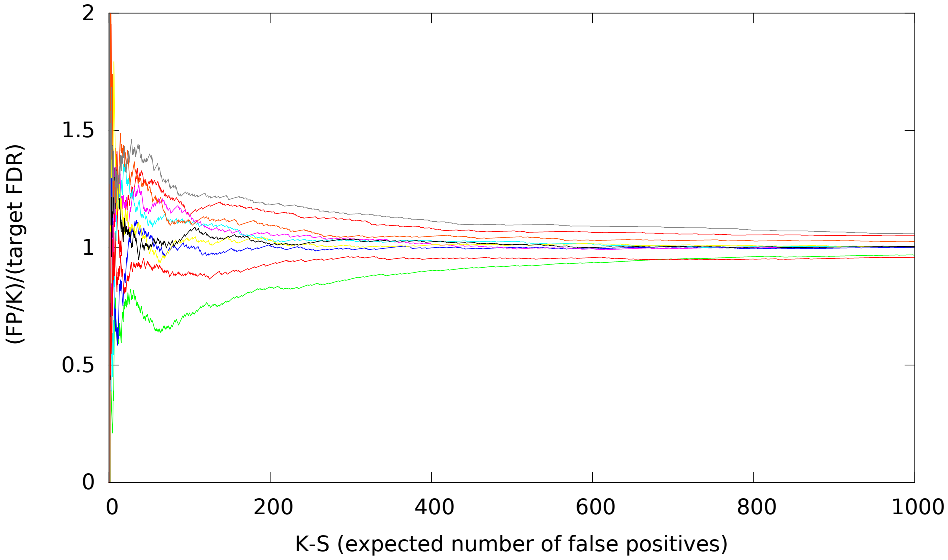

Figure 8: The variablity in the proportion of false positives vanishes as $K - S$ grows
